# An Enhanced Hyper-Parameter Optimization of a Convolutional Neural Network Model for Leukemia Cancer Diagnosis in a Smart Healthcare System

**DOI:** 10.3390/s22249689

**Published:** 2022-12-10

**Authors:** Joseph Bamidele Awotunde, Agbotiname Lucky Imoize, Oluwafisayo Babatope Ayoade, Moses Kazeem Abiodun, Dinh-Thuan Do, Adão Silva, Samarendra Nath Sur

**Affiliations:** 1 Department of Computer Science, Faculty of Information and Communication Sciences, University of Ilorin, Ilorin 240003, Nigeria; 2 Department of Electrical and Electronics Engineering, Faculty of Engineering, University of Lagos, Akoka, Lagos 100213, Nigeria; 3 Department of Electrical Engineering and Information Technology, Institute of Digital Communication, Ruhr University, 44801 Bochum, Germany; 4 Department of Computing and Information Science, School of Pure & Applied Sciences, College of Science, Bamidele Olumilua University of Education, Science & Technology, Ikere-Ekiti 361264, Nigeria; 5 Department of Computer Science, Landmark University, Omu-Aran 251103, Nigeria; 6 Department of Computer Science and Information Engineering, College of Information and Electrical Engineering, Asia University, Taichung 41354, Taiwan; 7 Instituto de Telecomunicações (IT) and Departamento de Eletrónica, Telecomunicações e Informática (DETI), University of Aveiro, 3810-193 Aveiro, Portugal; 8 Department of Electronics and Communication Engineering, Sikkim Manipal Institute of Technology, Sikkim Manipal University, Majitar, Rangpo 737136, Sikkim, India

**Keywords:** Internet of Medical of Things, convolutional neural network, deep learning, machine learning, diagnosis, leukemia dataset, prostate cancer dataset, hyper-parameters

## Abstract

Healthcare systems in recent times have witnessed timely diagnoses with a high level of accuracy. Internet of Medical Things (IoMT)-enabled deep learning (DL) models have been used to support medical diagnostics in real time, thus resolving the issue of late-stage diagnosis of various diseases and increasing performance accuracy. The current approach for the diagnosis of leukemia uses traditional procedures, and in most cases, fails in the initial period. Hence, several patients suffering from cancer have died prematurely due to the late discovery of cancerous cells in blood tissue. Therefore, this study proposes an IoMT-enabled convolutional neural network (CNN) model to detect malignant and benign cancer cells in the patient’s blood tissue. In particular, the hyper-parameter optimization through radial basis function and dynamic coordinate search (HORD) optimization algorithm was used to search for optimal values of CNN hyper-parameters. Utilizing the HORD algorithm significantly increased the effectiveness of finding the best solution for the CNN model by searching multidimensional hyper-parameters. This implies that the HORD method successfully found the values of hyper-parameters for precise leukemia features. Additionally, the HORD method increased the performance of the model by optimizing and searching for the best set of hyper-parameters for the CNN model. Leukemia datasets were used to evaluate the performance of the proposed model using standard performance indicators. The proposed model revealed significant classification accuracy compared to other state-of-the-art models.

## 1. Introduction

Due to internal and structural changes in organs, both with and without cause, the medical industry is currently dealing with several issues [1]. Healthcare specialists determine the cause of alterations in tissue, organs, and functionalities of the patient at the initial stage [2]. Using standard diagnosis procedures for several diseases, including high blood pressure and temperature changes, various types of cancer, heart attack, genetic disease, chronic disease, and hereditary disease, among others, are becoming difficult to diagnose and predict [3]. Although some sicknesses are difficult to predict early due to a lack of symptoms, it is still possible to track slight alterations in a person’s body [4,5]. The internal alterations of the human body must then be continuously observed to detect sickness in the early stages. The Internet of Medical Things (IoMT) is a network of devices used to gather data by attaching small devices to the bodies of patients to obtain information [6,7,8]. According to research carried out in 2017, there are now 8.4 billion IoT devices in use, and by 2020, there will be 30 billion [9,10]. IoMT devices have been successfully used in the medical industry to record the patient’s activity because of their effectiveness in capturing patient physiological signs [11]. An IoMT healthcare device is a tiny chip inserted into a watch, clothing, or similar item that is attached to a transmission device and gathers data based on the sensor [12]. In this study, IoMT devices were been used to gather data on cancer, including changes in breast, skin, lung, and dental tissues, along with other abnormalities [13]. Due to issues that arise from the illness, the worst ailments increase the incidence and mortality of cancer [14]. Wearable medical IoT devices efficiently track individual changes in the human body without causing rashes or allergies [15].

**Figure 1:**
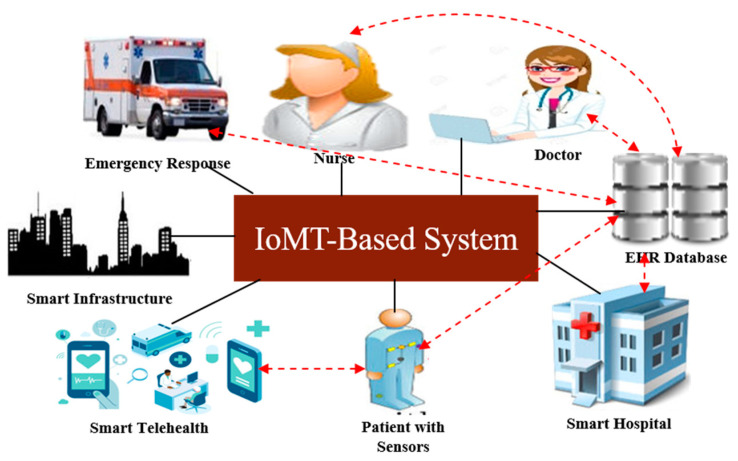
IoMT-based interventions in a smart healthcare system.

For instance, the wearable iTbra IoT gadget caught 50% of tissues linked to breast cancer that were previously used to forecast the disease and also predicted 17.3% of dense tissue without failing [16]. By using machine learning (ML) algorithms, 70 biopsies have been identified from the generated iTbra IoT data [17]. Dental tissue changes are captured by a small sensor chip in addition to the ITbra IoT device and can forecast cancer based on the patient’s everyday routines, habits, oral health, cavities, and other data [18]. Additionally, a skin-implantable, non-invasive, skin cancer diagnosis device that was created utilizing a field-programmable gate array application process [19] can capture rashes, skin tissue alterations, and skin changes, and it uses machine learning approaches to potentially diagnose skin cancer [20,21]. Figure 1 illustrates a few IoMT medical interventions in light of the explanation above. The mortality rate around the world is significantly impacted by various cancers, which are all lethal diseases.

### 1.1. Motivation

The abnormal proliferation of cancerous cells in the patient’s body causes cancer to spread quickly. Early cancer identification can enhance patient overall survival and medical interventions. For the accurate diagnosis and treatment of cancer, several screening techniques using computer-aided diagnosis and prediction techniques have been proposed. The DL-based model is used to extract features from datasets related to cancer, since DL approaches can extract features faster and more correctly than other methods currently in use. DL-based models effectively support currently used techniques, such as biopsy and mammography screening, when assessing and identifying breast cancer. In this study, a cloud-based methodology used for autonomous breast cancer stage diagnosis enabled by the Internet of Medical Things (IoMT) is proposed. An ML method can be implemented to assess IoMT medical data and anticipate pathological effects on the human body due to the difficulty of making decisions for a certain disease [22,23]. The standard ML-based method can be used to properly evaluate the IoMT-based captured features. However, it is challenging to accurately predict anomalous patterns, and including attributes adds complexity [24].

Therefore, this study introduces the convolutional neural network (CNN) with optimized features using hyper-parameter optimization for the diagnosis and prediction of cancer and support of disease-related psychological choices. IoMT-based data analysis includes examining the features of the collected data using particle swarm optimization (PSO) feature selection, which uses linked data to choose the optimum features both locally and globally. The dimension of the feature set is efficiently reduced through this PSO feature selection method. Intellectual aberrant patterns are categorized from the chosen features; therefore, the offered strategies efficiently handle disease-related decisions by utilizing the aforementioned described classifiers.

The main intention of this research work was to create a diagnostic model based on IoMT that can properly diagnose patients with cancer and healthy individuals. To classify patients with malignant and benign cancer, an optimized hyper-parameter CNN model was applied. The PSO approach was used to select features that improved the model’s performance. The PSO’s global search capability, resistance to control parameters, and computational effectiveness made it an appropriate choice for this study. By overcoming some feature selection biases to distinguish between the two kinds of cancer cells, benign and malignant, the proposed method varies from other studies. PSO was used to select the appropriate features in this process. The classification was performed using collected data from patients using IoMT-based sensors and devices. The IoMT-based diagnostic system based on the DL-based model was proposed to improve the classification accuracy of the IoMT-based platform. According to the literature review, current research has focused on hyper-parameter optimization rather than utilizing classification classifiers alone [24].

### 1.2. Contribution

The proposed model was developed using Python programming language with the collected information. One of the top programming languages for AI, ML, and DL-based models is Python. High-level data structures, dynamic typing, dynamic binding, and a host of other features make it ideal for developing sophisticated applications. Its simplicity, scalability, and advanced security features make it the most suitable programming language for this study, in addition to the presence of a comprehensive library. Subsequently, the effectiveness of the proposed model was evaluated using precision, recall accuracy, F-measure, and mean absolute error rate performance measures. Compared to current approaches, this study’s proposed strategies and approaches are more efficient, since they enhance and optimize the selection of relevant parameters that help the DL model diagnose cancer in its early stages.

The following achievements and contributions are made by this study:

- the utilization of PSO for feature selection to be able to remove irrelevant parameters from the datasets used.
- a brand new framework for hyper-parameter optimization of the CNN model to produce the best classification outcomes.
- the proposed model is simple to implement and can be used to accurately diagnose cancer in the IoMT-based healthcare environment.
- an experimental comparison of the proposed model with state-of-the-art classifiers that have been trained and evaluated using the same dataset.

### 1.3. Organization

The remaining paper is organized as follows: Section 2 presents the related work on cancer prediction. Section 3 presents the proposed IoMT-based framework model for leukemia diagnostics and classification; the hyper-parameter optimization for the CNN model is explained in detail, as is the CNN model and the PSO feature selection algorithm, along with the performance evaluation used for the testing and comparison of the proposed model. Section 4 presents the results analysis of the proposed model with comparison of the proposed model to other state-of-the-art classifiers using the same dataset for experimental analysis. Section 5 discusses the key findings and implications of the results. Finally, Section 6 presents the conclusion with recommendations for future research.

## 2. Related Work

IoMT is one of the networks that is presently advancing the quickest and is responsible for acquiring and using sensors in a medical setting to communicate enormous amounts of data [25]. In the healthcare field, IoT, also known as IoMT or medical IoT, is regarded as an expert application [26,27]. The term “IoMT” describes a networked architecture of medical software, hardware, platforms, processes, protocols, and communications. Using clever portable devices, sensor nodes on the patient’s body collect data to assess the patient’s physical characteristics [28]. IoMT enables secure internet connections for remote and wireless devices, while the application of AI algorithms provides speedy and flexible analysis and diagnosis of medical data. IoT devices manage a variety of unclear variables when transporting data via the cloud, including network architecture, energy transmission, and processing power [29]. Patients and caregivers alike have satisfactorily acclimated to remote monitoring of patients, and diseases can be detected and effectively treated using telehealth services. The shift to Industry 4.0 in healthcare is made possible by all of these applications and platforms [30].

The DL-based model is an ML algorithm endeavor that can be applied to automatically train and select models using datasets that include features of various cancer [31]. Many studies have made use of leukemia [32,33,34], prostate cancer [35,36,37], and other non-cancerous datasets for the prediction and classification of patients living with cancer, and ML-based models have been used for the diagnosis, prediction, and classification of these diseases, including Naïve Bayes (NB), logistic regression (LR), decision tree (DT), random forest (RF), and support vector machine (SVM) classifiers, among others. Various feature selection techniques have been used to improve the prediction accuracy of several classifiers through the application of best features during classification, such as bio-inspired algorithms, embedding, filter, and wrapper models [38].

The suggested CNN intervention for breast cancer classification (CNNI-BCC) model has helped medical professionals detect breast cancer, according to a study conducted by authors in ref. [39]. The suggested method categorizes different forms of breast cancer using supervised deep-learning neural networks. Data from 221 actual patients showed 90.50% accuracy in the results. Without any background experience, this model intelligently classified and detected breast cancer tumors, demonstrating an improvement over earlier techniques. Examination of the model showed that it was capable of analyzing the circumstances of impacted patients during the detection procedure.

According to the authors in ref. [40], CAD is diagnosed by addressing a variety of tissue irregularities. To automatically detect breast cancer, the researchers developed a CAD model based on a deep belief network (DBN) and divided breast regions into those that were healthy, benign, and cancerous. In light of the relevant fields of interest, two methods were introduced, with the initial approach designed for a small, predicted target. When the entire bulk was being targeted, the second strategy was used. The suggested model was trained and tested using a total of 347 images. The accuracy of the proposed CAD model for the two methods was 92.86% and 90.84%, respectively. When compared to other CAD systems currently in use, the results demonstrated increased efficiency.

ML research by the authors in ref. [41] has been shown to be quite useful in the field of healthcare. Handling the large data influx is complicated, and efficient management tools are needed. Deep learning techniques need a lot of data, which can be used in a variety of ways to achieve reliable results, and are crucial for applications in medicine. Medical datasets have various problems, including insufficient data, little sampling, inefficiencies in sampling, and challenges with large-scale applications. Ahmed et al.’s research utilized several learning approaches, a sizable dataset of medical images, and transfer learning, which were developed using a small dataset. The study’s classification of breast cancer, division of malignant regions, and pattern extraction from mammograms served as its foundation. For the suggested model, mammography images were subjected to preprocessing, such as noise removal, and feature extraction removed superfluous data items. The dataset from breast ultrasounds was classified and segmented using CAD. Using a pre-trained classifier and the transfer learning approach, image classification was carried out, after which each image was classified as either malignant or not. Finally, the tumor region in the afflicted photos was located using the R-CNN technique.

The existing research has some significant flaws, including poor classification pinpoint accuracy for advanced-level cancer and disregard for binary classes. More effective network models are still required for precise cancer region localization to aid in the early identification of various cancer [35,36,37,38,39]. To create an effective classifier for cancer classification, increasingly advanced cutting-edge networks and other CNN pre-trained models should be investigated [41,42,43,44,45,46,47,48,49,50,51,52,53]. The comparison of numerous hyper-parameter tuning techniques makes this study significant, and most crucially, the diagnosis of cancer is achieved, which is a classification problem. Numerous research models have addressed the leukemia classification issue using ML or DL techniques [38,39,40,41]. However, to our knowledge, none of these models have employed algorithms for hyper-parameter optimization to identify the best hyper-parameters, which result in the DL algorithm utilized in this study having the best classification performance. Nevertheless, depending on the classification issue, the optimized set of hyper-parameters is not universal.

## 3. Materials and Methods

**Figure 2:**
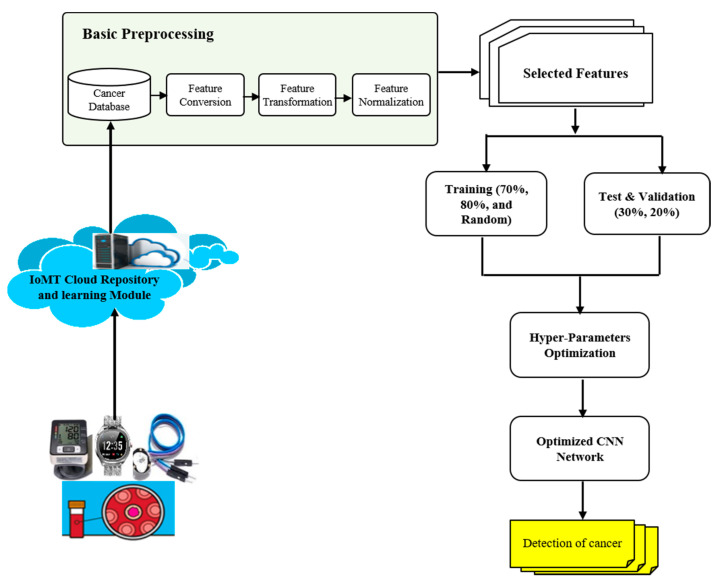
The proposed framework for the IoMT-based cancer diagnostics system.

Figure 2 displays the proposed framework for the IoMT-based cancer diagnostics architectural design using the proposed hyper-parameter-optimized CNN classifier.

### 3.1. Pre-Processing

The incoming data was first organized to create a dataset and analytical format. The gathered data can include values that are incorrect or missing, and additional details were whittled down via the normalization procedure to effectively remove noise from the dataset. An algorithm for numerical scaling normalization was used to process the collected data, which effectively deleted the data from the dataset. This technique analyzed any quantity of data in the datasets (data in small, medium, and big volumes) and effectively scaled the dataset from 0 to 1. The normalization procedure proceeded as follows (1):

(1) $$\frac{ND=\left(\left|X\right|-\left(10^{n-1}\right)\;\times \;\left(\left|F\right|\right)\right)}{10^{n-1}}$$

In Equation (1), ***ND*** is expressed as data that has noise eliminated and has been scaled to a certain input value. Input value ***X*** is indicated, n is the number of integers in the specific input ***X***, and the element’s first digit, ***F***, is used to symbolize it. For every integer value found in the captured IoMT-based cancer data, this technique was continually performed.

### 3.2. Feature Selection Using Particle Swarm Optimization

The use of feature selection is crucial since it can increase the classification accuracy, accelerate prediction, and decrease overfitting [43,44]. PSO-selected feature sets were used in the two-stage classification to distinguish whether a patient had cancer or not. The PSO approach was employed since it was likely that using the optimizer would improve the performance of the suggested method. Because it would be extremely expensive to test every possible scenario in a complete factorial fashion, the PSO was used to optimize the hyper-parameters. PSO is an algorithm for problem-solving that makes use of a population of potential solutions, known as particles. Based on their position and velocity, individual particles are distributed using a simple mathematical process around the search region. The local best-known location of each particle affects its motion, yet it is also directed toward the most well-known positions in the search area, which are upgraded when other particles find better locations. This will drive the swarm to move toward the best options [45].

The multidimensional search space contains a collection of m particles. The i–th particle’s position and velocity in the t–th iteration is $X_{i,t}$ and $V_{i,t}$, respectively. The particle modifies its position and speed by regulating two perfect solutions. The first is the desired result that the particle itself wants to achieve, specifically, the most intimate best $pbest_{i}$. The group is currently pursuing the alternative as the best course of action, perhaps the global $gbest_{t}$. Two mathematical equations are used in PSO to update the positions of each mass partner in the global search space, as indicated in Equations (2) and (3). In Equation (2), the coefficients $c_{1}$ and $c_{2}$ and random integers $r_{1}$ and $r_{2}$ are used, each possessing a location in the searching space of $x_{i}$ on $R^{n}$ and a velocity of $v_{i}$ on $R^{n}$.

(2) $$v_{i}^{k+1}=v_{i}^{k}+c_{1}r_{1}\left(pbest_{i}^{k}-x_{i}^{k}\right)+c_{2}r_{2}\left(gbest-x_{i}^{k}\right)$$

(3) $$x_{i}^{k+1}=x_{i}^{k}+v_{i}^{k+1}$$

Compared to mathematical algorithms and other heuristic optimization techniques, the PSO algorithm has the following primary benefits: a straightforward concept, straightforward implementation, robustness to control parameters, and computational efficiency. Similar to other heuristic optimization approaches, PSO is a derivative-free method. In comparison to more traditional mathematical methodologies and other heuristic techniques, PSO is less sensitive to the characteristics of the objective function [54,55]. Compared to other competing heuristic optimization methods, PSO contains fewer parameters, including only the inertia weight factor and two acceleration coefficients. Additionally, compared to other heuristic algorithms, the impact of the parameters on the answers is thought to be less sensitive [56]. In comparison to other stochastic approaches, PSO techniques can produce high-quality solutions with stable convergence characteristics in less time [57]. In comparison to other evolutionary methods, PSO appears to be somewhat less dependent on a set of initial points, suggesting that the convergence algorithm is reliable.

### 3.3. Hyper-Parameter Optimization

Different hyper-parameters utilized to control the structure and learning process of the network are dependent on neural networks, which can be categorized as computational and structural hyper-parameters [46]. The network’s architecture and structure are indicated based on the number of network layers, transfer function, degree of connectivity, neurons in each layer, and other structural hyper-parameters. Because they alter the structure of the network, the hyper-parameters affect its effectiveness and computational complexity, the learning approach, training dataset size, and other algorithmic parameters govern learning, velocity, rate of learning, etc. Hyper-parameters, which are not included in the model, have no impact on how well the neural network model performs. However, they have an impact on the training stage’s performance and pace.

For DL models, hyper-parameter settings are a collection of predetermined choices that directly affect the learning process and output of the prediction, which demonstrates how effectively the model learns and trains. The model is trained to search for patterns in a dataset and these patterns are used to train the model to predict the outcomes of incoming data. The selection of hyper-parameters is directly impacted by model design, which highlights the complexity of the model, and the time required to create and evaluate the model. Due to the uncertainty surrounding the ideal selection of parameters and the fact that they determine how well a model works, the setting has been a crucial and challenging subject in the use of DL algorithms.

Hyper-parameters are significant for DL-based models because they have a direct impact on controlling the behavior of the training model, hence contributing to the model’s high performance. Based on the scientist’s awareness, a manual search determines the hyper-parameter value and can be used if the researcher has a firm grasp of neural network topology and learning data. However, the standards for choosing hyper-parameters are ambiguous, calling for several experiments. In this study, the hyper-parameter optimization through radial basis function and dynamic coordinate search (HORD) algorithm was used on each hyper-parameter, and several values were computed and combined to arrive at the hyper-parameters used. HORD is very effective and simple when it comes to finding the best hyper-parameters for the CNN classifier. With HORD, all combinations of hyper-parameter values were investigated using the top and lower boundaries of each hyper-parameter to determine the ideal values and a predetermined step size for the variable range of each hyper-parameters was established.

HORD was introduced as a more effective method because it uses a deterministic model [54], unlike other optimization approaches. By using certain starting sample points and the radial basis function (RBF) approximation, the deterministic model employed in this method was produced as follows (4):

(4) $$S_{t}\left(h\right)=\sum_{d=1}^{t}{⋌}^{\left(d\right)}{(||h-h^{\left(d\right)}||)}^{3}+p\left(h\right)$$

where $||.||$ denotes the interpolation parameters and ⋌p is the Euclidean norm [54]. Upon creation of the model, the perturbation $\delta_{d}$ is used to produce candidates $h_{cand}^{\left(1:c\right)}$ based on the top-performing observation $h_{test}$ that adheres to a particular normal distribution. The following is the definition of the probability of perturbation $\varphi_{t}$ (5):

(5) $$\varphi_{t}=\varphi_{0}\left[1-\frac{ln\left(t-t_{0}+1\right)}{ln\left(N_{max}-t_{0}\right)}\right],\;t_{0}\;\leq t<N_{max}$$

where t is the algorithm’s iterations, $t_{0}$ is the number of initial observations used to fit the model, and $N_{max}$ is the algorithm’s maximum number of iterations. D is the total number of dimensions for the hyper-parameters and the value of $\varphi_{0}$ is set to $min\;\left(20/D,\;1\right)$.

Subsequently, using the candidates generated, the following formula is used to determine the final weighted score (6):

(6) $$W\left(h_{cand}\right)=\epsilon W^{cv}\left(h_{cand}\right)+\left(1-e\right)W^{dm}\left(h_{cand}\right)$$

The two criteria are added together to determine the final weighted score, where $W^{cv}$ is the measure used to determine how well the surrogate models are estimated in Equation (7), the distance metric represented by $W^{dm}$ is calculated in Equation (8), and each criterion’s weight is indicated by ϵ.

(7) $$W^{cv}\left(h_{cand}\right)=\left\{\begin{array}{c} \frac{S_{max}\;-\;S\left(h_{cand}\right)}{S_{max}\;-\;S_{min}},\;if\;S_{max}\;\neq \;S_{min}\;\\ 1,\;\;\;\;\;\;\;\;\;\;\;\;\;\;\;\;\;\;\;\;\;\;\;\;\;\;\;\;otherwise \end{array}\right.$$

where $S_{max}=\max\left\{S\left(h_{cand}\right)\right\},\;S_{min}=\min\left\{S\left(h_{cand}\right)\right\}.$

(8) $$W^{dm}\left(h_{cand}\right)=\left\{\begin{array}{c} \frac{\Delta_{max}\;-\;\Delta \left(h_{cand}\right)}{\Delta_{max}\;-\;\Delta_{min}},\;if\;\Delta_{max}\;\neq \;\Delta_{min}\;\\ 1,\;\;\;\;\;\;\;\;\;\;\;\;\;\;\;\;\;\;\;\;\;\;\;\;\;\;\;\;\;\;otherwise \end{array}\right.$$

where $\Delta \left(h_{cand}\right)$ denotes the separation between previously assessed positions $h_{1:t}$ derived from $\Delta \left(h_{cand}\right)=\mathrm{mn}\|h_{cand}-h_{1:t}\|$ = minhcand-$h_{1:t}$. Then,

$$\Delta_{max}=\max\left\{\Delta \left(h_{cand}\right)\right\},\;\Delta_{min}=\min\left\{\Delta \left(h_{cand}\right)\right\}.$$

Finally, the hyper-parameter set for the following step $h^{*}$ is discovered using the final weighted score, and the surrogate model $S_{t}\left(h\right)$ is continuously updated since the genuine neural network model evaluates $h^{*}$. Algorithm 1 shows the HORD hyper-parameters used to select the optimal parameters for the CNN model.

**Algorithm 1:** HORD Algorithm.
*1: Generate a little insight*

$A_{t0}=\;{\left\{h^{\left(d\right)},\;G\left(h^{\left(d\right)}\right)\right\}}_{d=1}^{t_{0}}$

*measuring with Latin hypercubes;*

*2: while*

$t\;<N_{max}$

*do*
*3: Fit or revise the*$S_{t}\left(h\right)$*RBF interpolation model from (4) using*$A_{t}$.
*4: Fix*

$h_{test}=argmax\left\{G\left(h\right)\right\}\;in\;A_{t}$

*;*

*5: Create c candidates using*

$h_{cand}^{\left(1:c\right)}$

*based on*

$h_{test}$

*and*

$\delta_{d}$

*samples were taken from a normal distribution with a certain probability*

$\varphi_{t}$

*in (5).;*

*6: Calculate*

$W_{t}^{cv}h_{cand}^{\left(1:c\right)}$

*by (7),*

$W_{t}^{dm}h_{cand}^{\left(1:c\right)}\;by\;\left(8\right)$

*, as well as the final weighted score*

$W_{t}h_{cand}^{\left(1:c\right)}$

*by (6).*

*7: Set*

$h^{*}=argmin\left\{W_{t}h_{cand}^{\left(1:c\right)}\right\}$

*.*

*8: Estimate*

$G\left(h^{*}\right)$

*.*

*9: Update*

$A_{t+1}=\;\left\{A_{t}\;\cup \left(h^{*},\;G\left(h^{*}\right)\right)\right\}$


*10: **end while***
*11: Find*$h_{test}=argmax\left\{G\left(h\right)\right\}\;in\;A_{N_{max}}$.*12: Return*$h_{test}$.

The ideal hyper-parameter values for ML algorithms are chosen using designs of experiment (DOE) methods [47]. DOE evaluates the effects of numerous experimental components simultaneously, with each experiment comprising several runs with various hyper-parameter settings that should be evaluated collectively. After the trials are finished, the experimental results are statistically examined to ascertain how the hyper-parameters affect the performance of the classifiers. To put it differently, a model is created that empirically connects classification performance, such as incorrect predictions (as a reaction parameter), to hyper-parameters (as indicators of classifier effectiveness). Table 1 lists the hyper-parameters adjusted for the proposed CNN model.

### 3.4. Convolutional Neural Networks (CNNs)

CNN is a well-liked deep-learning technique for image analysis. Convolution is a type of computation where two functions are combined to create a third function, which is defined as the product of two functions after a variable has been shifted and inverted. In CNN, an array of weights known as filters is created when the input is subjected to a convolution, which results in the creation of an object map. At each time step, the filter passes across the input while multiplying the matrix. Each entity (input parameter) is given this treatment, and the outcomes are blended to provide a new collection of chosen features. Dilating causal convolutions are frequently employed in the context of series or time series. Causality suggests that the filter’s output is independent of incoming time steps in the event. By stacking dilated convolutions, the network can retain input scale while looking back in time with fewer layers (i.e., how many time steps there are in the sequence) and computing effectiveness. As the network depth increases, each additional layer exponentially raises the dilation factor. The neural network’s epoch number indicates how many times it has gone through the training dataset. The network learns to make predictions more accurately as it is exposed to more data. On the other hand, excessive exposure can lead to overfitting. In this case, the training error is minor, but the error keeps on increasing as fresh data are presented. This increasing error can be stopped during data training any time the validation error is minimized and stops decreasing. During optimization, early blocks are used to hasten network learning.

The CNN design includes dropout layers, batch normalization, and one-dimensional convolution. A dense, completely linked layer utilized for categorization makes up the top layer, and the network weights are altered upon each batch. The completion of the training period occurs when all batches have traversed the network once. The loss function is used to assess how well the network matches the data, which is reduced throughout training by selecting the appropriate weights for the neurons. HORD is an optimization algorithm that is used to explain how the weights of neurons change as learning progresses. The learning rate is the maximum permitted variation in each stage of the training process in terms of the number of neurons. Excessive weight updates might result from a high learning rate, causing the network performance to vary during training epochs. A sluggish learning algorithm has the risk of failing to converge or becoming stuck in a poor outcome. The learning rate should therefore be calibrated. The quantity of data the neural network processes in a single phase is referred to as the batch size. As the batch size grows, more RAM may be required during the training phase.

### 3.5. The Description of the Leukemia Dataset

A wide variety of various malignancies are attracted to blood cancer, including lymphatic system and bone marrow malignancies. Bone marrow becomes more active in leukemia, which may impact its capacity to generate platelets and healthy white blood cells. These hematopoietic stem cell tumors are dangerous. Data on leukemia and cancer were obtained from the UCI repository. A total of 7129 genes were encountered and 72 samples were analyzed, all of which were collected from patients with acute leukemia, either acute myelogenous leukemia (AML) or acute lymphoblastic leukemia (ALL). In actuality, there were 25 cases of AML and 47 cases of ALL. The remaining data in the dataset contained cases of chronic myelogenous leukemia (CML), and chronic lymphocytic leukemia (CLL). The dataset had already undergone some normalization. Ratios were used to divide the dataset into training and testing sets. The dataset was divided into 70% to 30% and 80% to 20% partitions for the training and testing (validation) sets, and at random, in the proposed technique. The dataset was divided into two different sets to determine how well the model worked for the two partitions. Table 2 gives a detailed description of the leukemia datasets, divided into partitioned training and testing (validation) sets and at random in the proposed technique.

In the bone marrow or lymphatic system, lymphoid cells can become cancerous and progress into leukemia. This most frequently affects white blood cells, which makes it more challenging for the immune system to combat illness. Leukemia may be discovered accidentally during a physical examination or as a consequence of normal blood tests because many kinds of the disease do not manifest any evident symptoms early on. A doctor should consider leukemia if a patient has pale skin, enlarged lymph nodes, swollen gums, an enlarged liver or spleen, severe bleeding, bruises, fever, ongoing infections, exhaustion, or a small pinpoint rash. An abnormal white cell count on a blood test may point to the diagnosis. A needle biopsy and aspiration of bone marrow from a pelvic bone is required to confirm the diagnosis and determine the precise kind of leukemia. The bone marrow will be examined for leukemic cells, DNA markers, and chromosome abnormalities. Age, leukemia type, and chromosomal abnormalities discovered in leukemia cells and bone marrow are all significant factors in leukemia. From the leukemia dataset, 2323 genes among 7129 genes (31.59% of the genes) were chosen for the proposed model classification.

### 3.6. The Performance Evaluation Metrics Used to Evaluate the Proposed Model

To assess the effectiveness of the model, six assessment metrics were used. True positive (TP) indicated that a person had the disease; true negative (TN) denoted a healthy individual; false positive (FP) denoted the diagnosis of leukemia in a healthy individual; and false negative (FN) referred to the classification of a breast cancer patient as benign. Equations (9)–(13) explain the performance metrics used in the proposed framework.

The performance of the classification system is shown by classification accuracy (CA) given in Equation (9):

(9) $$CA=\frac{TN+TP}{The\;total\;number\;of\;test\;items}$$

Recall is defined as the ratio of accurately predicted positive occurrences to all actual positive occurrences in the class, given in Equation (10):

(10) $$Recall=\frac{TP}{TP+FN}*100\%$$

Specificity demonstrates that a forecast is incorrect and that the subject is in good health, as defined in Equation (11):

(11) $$Specificity=\frac{TN}{TN+FP}*100\%$$

The harmonic mean of recall and precision is known as the F1-Score, given in Equation (12):

(12) $$F1-Score=\frac{precision*recall}{precision+recall}*100\%$$

Precision is the accuracy by which a condition is correctly identified by the model, given by Equation (13):

(13) $$Precision=\frac{TP}{TP+FP}*100\%$$

## 4. Experimental Results

Several evaluation criteria, including accuracy, sensitivity, specificity, recall, precision, and ROC, were utilized to assess the performance of the model. All implementation results are also shown in tables and graphs for easier interpretation. An HP Core i5 with 8 GB RAM and a 2.0 GHz processor running on Windows 10 Operating System was used for all experiments.

The model was developed using Python 3.9.10 with the Keras 2.9.0 library and Tensor Flow 1.15 as the back end. On average, 25 s were needed to finish each period. Pylearn2 is an open-source ML-based library with an emphasis on DL techniques. It also promotes the use of GPUs, which can considerably speed up the execution of DL-based models. When there is sufficient data, DL-based NNs operate at their best.

Table 1 lists the hyper-parameters for the CNN design (number of layers, maximum pooling size for each layer, and kernel). Radial basis function and dynamic coordinate search were employed in the proposed model to optimize the hyper-parameters for the dataset. The hyper-parameters were used for which the model performed best on the leukemia dataset. Here, the CNN layer achieved leukemia classification while the objective dataset was updated to include each DL network’s taught and visually different characteristics by distributing an equal number of neurons between the two groups, since these fine-tuned parameters were not self-trained. It was essential to modify the optimal parameters following the outcomes of the training genes for performance enhancement. The results of an experiment comparing the tuned CNN architectures on the baseline sample dataset are shown in Table 3.

### 4.1. Performance Results of the Proposed Model Using the Two Partitions of the Dataset

According to Table 3, the model’s performance was superior to that of classifiers using CNN and PSO + CNN on the dataset. The accuracies of the CNN and PSO + CNN classifiers were 95.8% and 97.6%, respectively. However, after feature selection using a hyper-parameter to create a hybrid approach with 36 attributes, the accuracy was 99.6% due to a 3.8% increase compared to the CNN classifier and 2.0% increase compared to the PSO + CNN classifier. Multiple tests were run to improve feature reduction and eliminate unnecessary properties from the dataset. The experiments were carried out three times to test the performance of the proposed model against CNN and PSO + CNN using the 70% to 30% and 80% to 20% partitions and at random.

The effectiveness of the proposed model with the PSO feature selection method was evaluated. The performance evaluation revealed that, when compared to outcomes without PSO, the proposed model with feature selection from PSO produced results with higher levels of accuracy. PSO + the proposed model outperformed the proposed model without the feature selection for the diagnosis of leukemia, according to a comparison of the two outcomes. Although the performance of the proposed model with and without PSO was very good, the feature selection algorithm greatly increased the accuracy of leukemia diagnosis, with 99.9% accuracy versus 96.0% accuracy for the proposed model without the feature selection.

Table 4 displays the proposed model’s prediction performance for ALL and healthy cases, revealing the accuracy to be 99.9% and 100%, respectively. The precision, recall, and F1 score were also 100% or 1.0. The prediction accuracy for CLL was 99.8%, the recall was 98.8%, the specificity was 100%, the F1-score was 99.8%, and the precision was 100%, respectively. The prediction accuracy rate for AML was 99.9%, and the precision, recall, and F1 score were 100%. The dataset was divided into 80% for training and 20% for testing (validation), respectively.

**Figure 3:**
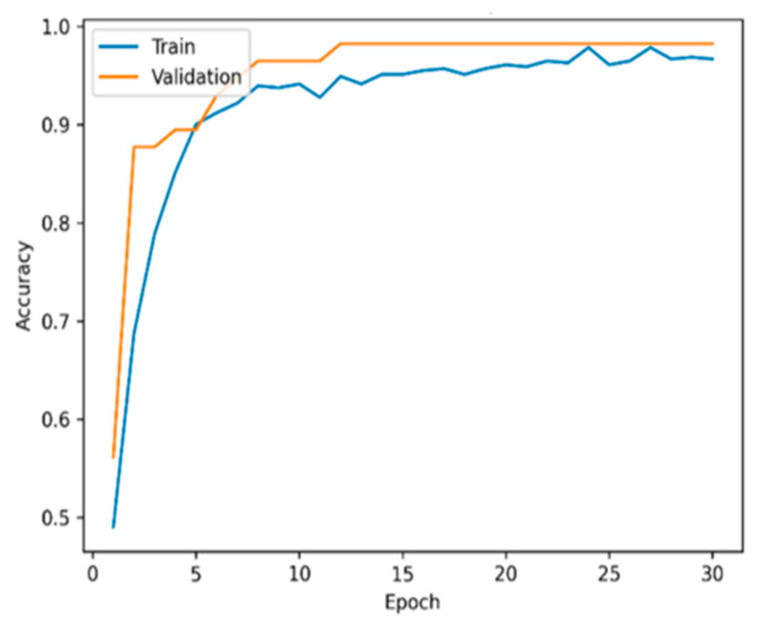
Model accuracy.

**Figure 4:**
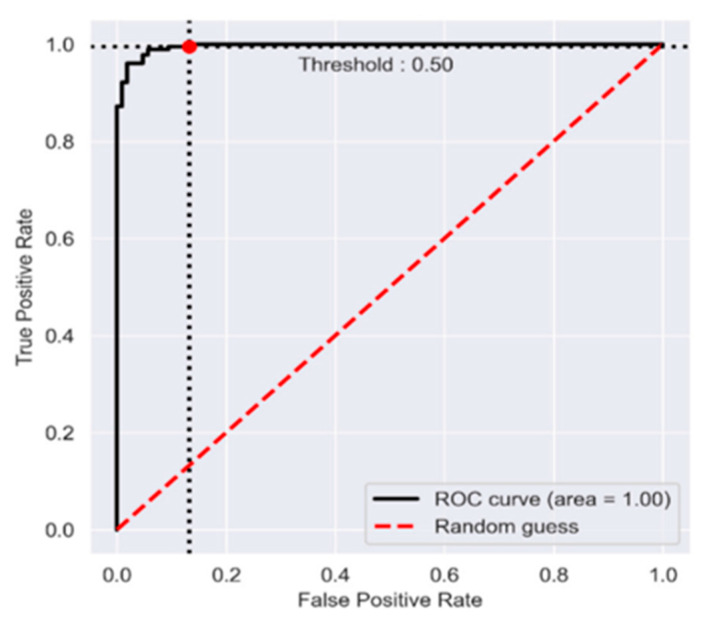
Receiver Operating Characteristic (ROC).

Figure 3 and Figure 4 display the model accuracy and ROC for the proposed model. Figure 3 shows that the proposed model greatly enhanced the performance of leukemia diagnosis. The proposed model had an AUC of 1.00, as shown in Figure 4.

### 4.2. Comparison of the Proposed Model with Other State-of-the-Art Models

The outcomes of other studies using the same datasets are shown in Table 4, which can be used for objective comparison. Compared to other existing models in the literature, the results showed that the proposed model achieved greater accuracy in the microarray datasets utilized for the performance measures. In general, the proposed model outperformed recently used deep learning methods, as well as traditional and hybrid machine learning methods. The outcomes showed that the proposed method may be used to select and categorize cancer-related genes from sparse datasets with accuracy and efficiency. The results further demonstrated the applicability of the proposed methodology for precise cancer subtype detection and diagnosis. Table 5 shows the comparison of the accuracy of the proposed study to that of some existing models using the same dataset.

These results showed that the suggested CNN model can accurately predict leukemia. Convolutional neural networks are therefore a great alternative to time-consuming standard ML models. Findings from the hyper-parameter tuning revealed that some combinations of parameters had a greater impact on the model’s performance than others. The proposed framework revealed that the effectiveness of prediction was significantly improved and impacted by the number of layers and filter width. The outcomes further proved that high performance was possible at all filter widths. Additionally, using multiple layers produced somewhat better performance than using just one layer, since it permitted the model to be more complicated; however, this also resulted in a longer training period. Training time was directly influenced by the filter’s breadth and the number of layers, but had no impact on classification performance. Therefore, a high filter width required less training time than a smaller filter width if the number of layers was fixed, even though both options have the same forecasted results.

## 5. Discussion

Cancer is generally considered to be a high-risk disease globally, especially leukemia. Hematologists must recognize the presence of leukemia and its specific type to minimize medical risks and choose the best leukemia therapy. The detection of leukemia with an optical blood smear examination under a specialist’s supervision is an important and time-consuming procedure. To address such issues, various ML and DL techniques have been proposed for the diagnosis, prediction, and classification of peripheral blood mononuclear cells. However, these methods need to be improved in terms of the learning process, effectiveness, consistency, and classification accuracy. Therefore, to overcome some of these challenges and keeping the real-time vitality of healthcare in mind, this study proposed an IoMT-based framework for automatic diagnosis and classification of leukemia subtypes. In the proposed model, IoMT-enabled devices and sensors were used to capture various signs and symptoms from patients to the leukemia cloud. PSO feature selection was used to select relevant features that improved the classification performance, and hyper-parameter-optimized CNN was used for the diagnosis and classification of leukemia according to its types.

The proposed model had an AUC of 1.00, according to the receiver operating characteristics. After diagnosis and classification of cancer using the proposed model, the information is transferred to the physician’s device (computer or smartphone), where the physician uses the IoMT infrastructure to continue providing medical care based on the test results. The proposed system had better accuracy when compared with some state-of-the-art classifiers using the same dataset for performance evaluation. Authors in ref. [50] reported the second-best accuracy of 98.6% using the GSP model, authors in ref. [51] came third with an accuracy of 97.0% using the IG-SGA classifier, and the authors in ref. [52] has the lowest accuracy of 57.9% using the SEEIDCNN model. The proposed model also performed better across other performance metrics.

In a handful of ML scenarios, deep networks have already surpassed simplistic techniques, but this is not usually the case. A deficiency of a parameter match could be one such problem. The small size of the data could be another factor. To be adequately constructed, deep networks need considerably bigger training datasets because they are very highly dependent on the size of the training set. The findings of the proposed framework demonstrate that no particular set of hyper-parameters substantially surpassed the others. Due to adjustments to weight and bias initialization, it is not always the case that retraining a classifier with the same hyper-parameters will yield the same classification performance. As a result, it is essential to run training many times before selecting the best network. However, deeper networks with more layers often take longer to train.

Optimization of the hyper-parameter values remains the major benefit of the proposed model, as well as PSO to remove irrelevant features from the leukemia dataset. A crucial component of controlling the behavior of both ML- and DL-based models is hyper-parameter tuning. If the hyper-parameters are not properly set, the predicted model parameters produce inferior results, given that they do not reduce the gradient descent. Many hyper-parameters are frequently used in contemporary ML techniques (one to a thousand) and they are essential for transferability of the model. Professional expertise and understanding are required for this task. Furthermore, it takes a lot of time to conduct searches over fully developed hyper-parameter domains. Typically, the excitable search only trains a small number of potential setups over a short time, and usually the highest qualified candidates receive comprehensive training. It remains unclear how to create a brand new hyper-parameter optimization approach that combines all of the benefits of both automation and professional understanding. Therefore, future work will employ more refined and automated neural architecture search techniques to improve the proposed model and create a strong CNN classifier.

## 6. Conclusions

This study presents a hyper-parameter optimization of a CNN model for the early diagnosis and classification of leukemia. The hyper-parameter-optimized CNN model was used to diagnose and classify leukemia subtypes in the IoMT-based healthcare system, which collects data using various sensors and devices. The collected data that formed the dataset were initially analyzed through pre-processing techniques to replace missing values using the min-max method and relevant features were selected using the PSO technique. To identify differences in leukemia patterns, several variables connected to leukemia were extracted from the noise-free data and supplied to the classifier. The results of the proposed system revealed that the hyper-parameter-optimized CNN model enabled with PSO outperformed other state-of-the-art classifiers in the literature. The results revealed that the proposed model provided an accuracy of 99.9% and 100% across all of the tested performance metrics. Overall, the findings of the study indicate that the IoMT-based system enabled with PSO and hyper-parameter-optimized CNN was effective and valid for the successful real-time and smart diagnosis and classification of leukemia. However, the security and privacy of IoMT-based environments are paramount to being able to protect patient data and leukemia diagnosis results from an unauthorized user. Hence, future studies will consider the security and privacy of the proposed system to provide open network computing systems and communication in a secure environment.

## Figures and Tables

**Table 1:** The CNN model hyper-parameter settings and their ranges.

Hyper-Parameter	Explanation	Range
Neuron Count	The number of neurons in the top Convolutional layers	8, 16, 32
Layer Depth	The total number of layers in the network	1, 2, 3
Kernel Size	Size of the convolutional layer’s kernel	1, 2, 3
Stride	The quantity of shifting kernel pixels during convolution	1, 2, 3
Activation Function	The process of activating neurons	Sigmoid, ReLU, SeLU
Batch Size	Number of training data divisions per group	8, 16, 32
Kernel Count	Number of convolutional layer kernels	8, 16, 32
Epoch	Numerous iterations of learning	20, 50, 100
Learning Rate	Updated weight during learning	0.01, 0.001, 0.0001
Loss Function	A method for calculating error	L2 loss, Binary cross-entropy

**Table 2:** Detail description of the leukemia datasets.

Dataset	Number of Genes	Samples	Classes
AML-ALL	7129	72	2
AML-ALL-CML	7129	72	3
AML-ALL-CML-CLL	7129	72	4

**Table 3:** the performance comparison results in the leukemia dataset.

Feature Selection	Classifier Technique	Experiment	Accuracy (%)	Recall (%)	Specificity (%)	Precision (%)	F-Score (%)
None	CNN	(70-30)	95.2	95.1	94.8	95.3	95.4
		(80-20)	95.8	95.5	95.2	94.9	96.1
		Random	96.5	96.3	95.7	96.1	95.9
		Mean	95.8	95.6	95.2	95.4	98.8
PSO	CNN	(70-30)	97.0	97.1	98.0	97.9	95.2
		(80-20)	97.3	97.5	98.0	98.3	98.1
		Random	98.5	98.2	98.0	98.9	98.7
		Mean	97.6	976	98.0	98.4	97.3
PSO + Hyper-parameter	CNN	(70-30)	98.8	98.9	99.7	99.9	99.7
		(80-20)	99.9	99.9	99.7	99.9	99.7
		Random	100	99.9	99.8	100	99.9
		Mean	99.6	99.6	99.7	99.9	99.8

**Table 4:** The effectiveness of the suggested model for identifying leukemia subtypes.

Measures	Accuracy (%)	Recall	Specificity	F1-Score	Precision
ALL	99.9	1.0	1.0	1.0	1.0
AML	99.9	1.0	1.0	1.0	1.0
CML	100	1.0	1.0	1.0	1.0
CLL	99.8	0.98	1.0	0.99	1.0
Healthy	100	1.0	1.0	1.0	1.0

**Table 5:** The comparison of the accuracy of the proposed model on microarray datasets.

Methods	Authors	Dataset	Size of Dataset	Accuracy (%)
SVM-RFE + BDF	Medjahed et al. (2017) [48]	Leukemia	5147	95.8
AEN-CMI	Wang et al. (2019) [49]	Leukemia	7129	91.1
GSP	Alanni et al. (2019) [50]	Leukemia	5327	98.6
IG-SGA	Salem et al. (2017) [51]	Leukemia	7129	97.0
SEEIDCNN	Liu et al. (2017) [52]	Leukemia	12,600	57.9
Random Forest	Ram et al. (2017) [53]	Leukemia	22,283	95.2
PSO + Optimized CNN	Proposed Model	Leukemia	7129	99.9

Support Vector Machines Recursive Feature Elimination (SVM-RFE); Binary Dragonfly (BDF); Adaptive Elastic Net with Conditional Mutual Information (AEN-CMI); Gene Selection Programming (GSP); Information Gain (IG) and Standard Genetic Algorithm (SGA); Sample Expansion 1-dimensional Convolutional Neural Network (SE1DCNN).

## Data Availability

https://figshare.com/articles/dataset/The_microarray_dataset_of_leukemia_cancer_in_csv_format_/13658787, and https://www.kaggle.com/datasets/andrewmvd/leukemia-classification (accessed on 25 September 2022).

## References

[1] Kirubakaran J., Venkatesan G.K.D., Sampath Kumar K., Kumaresan M., Annamalai S. (2021). Echo state learned compositional pattern neural networks for the early diagnosis of cancer on the internet of medical things platform. J. Ambient. Intell. Humaniz. Comput..

[2] Awotunde J.B., Adeniyi E.A., Ajamu G.J., Balogun G.B., Taofeek-Ibrahim F.A. (2022). Explainable Artificial Intelligence in Genomic Sequence for Healthcare Systems Prediction. Studies in Computational Intelligence.

[3] Schneider P., Biehl M., Hammer B. (2009). Adaptive relevance matrices in learning vector quantization. Neural Comput..

[4] Baskar S., Shakeel P.M., Kumar R., Burhanuddin M.A., Sampath R. (2020). A dynamic and interoperable communication framework for controlling the operations of wearable sensors in smart healthcare applications. Comput. Commun..

[5] Awotunde J.B., Oluwabukonla S., Chakraborty C., Bhoi A.K., Ajamu G.J. (2022). Application of artificial intelligence and big data for fighting COVID-19 pandemic. International Series in Operations Research and Management Science.

[6] Awotunde J.B., Ayoade O.B., Ajamu G.J., AbdulRaheem M., Oladipo I.D. (2022). Internet of Things and Cloud Activity Monitoring Systems for Elderly Healthcare. Studies in Computational Intelligence.

[7] Nayyar A., Puri V., Nguyen N.G. (2019). BioSenHealth 1.0: A novel internet of medical things (IoMT)-based patient health monitoring system. International Conference on Innovative Computing and Communications.

[8] Dwivedi R., Mehrotra D., Chandra S. (2021). Potential of Internet of Medical Things (IoMT) applications in building a smart healthcare system: A systematic review. J. Oral Biol. Craniofacial Res..

[9] Awotunde J.B., Jimoh R.G., AbdulRaheem M., Oladipo I.D., Folorunso S.O., Ajamu G.J. (2022). IoT-based wearable body sensor network for COVID-19 pandemic. Stud. Syst. Decis. Control..

[10] Espinoza H., Kling G., McGroarty F., O’Mahony M., Ziouvelou X. (2020). Estimating the impact of the Internet of Things on productivity in Europe. Heliyon.

[11] Juneja S., Dhiman G., Kautish S., Viriyasitavat W., Yadav K. (2021). A perspective roadmap for IoMT-based early detection and care of the neural disorder, dementia. J. Healthc. Eng..

[12] Qureshi F., Krishnan S. (2018). Wearable hardware design for the internet of medical things (IoMT). Sensors.

[13] Awotunde J.B., Jimoh R.G., Folorunso S.O., Adeniyi E.A., Abiodun K.M., Banjo O.O. (2021). Privacy and security concerns in IoT-based healthcare systems. Internet of Things.

[14] Younossi Z.M. (2019). Non-alcoholic fatty liver disease–a global public health perspective. J. Hepatol..

[15] Legner C., Kalwa U., Patel V., Chesmore A., Pandey S. (2019). Sweat sensing in the smart wearables era: Towards integrative, multifunctional and body-compliant perspiration analysis. Sens. Actuators A Phys..

[16] Sridhar K.P., Baskar S., Shakeel P.M., Dhulipala V.R. (2019). Developing brain abnormality recognize system using multi-objective pattern producing neural network. J. Ambient. Intell. Humaniz. Comput..

[17] Manogaran G., Shakeel P.M., Hassanein A.S., Kumar P.M., Babu G.C. (2018). Machine learning approach-based gamma distribution for brain tumor detection and data sample imbalance analysis. IEEE Access.

[18] Yang B., Liao G.Q., Wen X.F., Chen W.H., Cheng S., Stolzenburg J.U., Ganzer R., Neuhaus J. (2017). Nuclear magnetic resonance spectroscopy as a new approach for improvement of early diagnosis and risk stratification of prostate cancer. J. Zhejiang Univ. Sci. B.

[19] Suresh A., Harish K.V., Radhika N. (2015). Particle swarm optimization over back propagation neural network for length of stay prediction. Procedia Comput. Sci..

[20] Baskar S., Shakeel P.M., Sridhar K.P., Kanimozhi R. Classification system for lung cancer nodule using machine learning technique and CT images. Proceedings of the 2019 International Conference on Communication and Electronics Systems (ICCES).

[21] Kumar R., Sampath R., Mohamed Shakeel P. (2020). Analysis of regional atrophy and prolonged adaptive exclusive atlas to detect Alzheimer’s neuro disorder using medical images. Multimed. Tools Appl..

[22] Awotunde J.B., Folorunso S.O., Bhoi A.K., Adebayo P.O., Ijaz M.F. (2021). Disease diagnosis system for IoT-based wearable body sensors with a machine learning algorithm. Hybrid Artificial Intelligence and IoT in Healthcare.

[23] Greco L., Percannella G., Ritrovato P., Tortorella F., Vento M. (2020). Trends in IoT-based solutions for health care: Moving AI to the edge. Pattern Recognit. Lett..

[24] Ghaderzadeh M., Asadi F., Hosseini A., Bashash D., Abolghasemi H., Roshanpour A. (2021). Machine learning in detection and classification of leukemia using smear blood images: A systematic review. Sci. Program..

[25] Mavrogiorgou A., Kiourtis A., Touloupou M., Kapassa E., Kyriazis D. (2019). Internet of medical things (IoMT): Acquiring and transforming data into HL7 FHIR through 5G network slicing. Emerg. Sci. J..

[26] Sodhro A.H., Pirbhulal S., Sangaiah A.K. (2018). Convergence of IoT and product lifecycle management in medical health care. Future Gener. Comput. Syst..

[27] Padikkapparambil J., Ncube C., Singh K.K., Singh A. (2020). Internet of Things technologies for elderly health-care applications. Emergence of Pharmaceutical Industry Growth with Industrial IoT Approach.

[28] Deebak B.D., Al-Turjman F., Aloqaily M., Alfandi O. (2019). An authentic-based privacy preservation protocol for smart e-healthcare systems in IoT. IEEE Access.

[29] Awotunde J.B., Misra S. (2022). Feature extraction and artificial intelligence-based intrusion detection model for a secure internet of things networks. Illumination of Artificial Intelligence in Cybersecurity and Forensics.

[30] Painuli D., Bhardwaj S. (2022). Recent advancement in cancer diagnosis using machine learning and deep learning techniques: A comprehensive review. Comput. Biol. Med..

[31] Abiodun M.K., Misra S., Awotunde J.B., Adewole S., Joshua A., Oluranti J. (2021). Comparing the Performance of Various Supervised Machine Learning Techniques for Early Detection of Breast Cancer. International Conference on Hybrid Intelligent Systems.

[32] Bibi N., Sikandar M., Ud Din I., Almogren A., Ali S. (2020). IoMT-based automated detection and classification of leukemia using deep learning. J. Healthc. Eng..

[33] Warnat-Herresthal S., Perrakis K., Taschler B., Becker M., Baßler K., Beyer M., Günther P., Schulte-Schrepping J., Seep L., Klee K. (2020). Scalable prediction of acute myeloid leukemia using high-dimensional machine learning and blood transcriptomics. Iscience.

[34] Boldú L., Merino A., Acevedo A., Molina A., Rodellar J. (2021). A deep learning model (ALNet) for the diagnosis of acute leukemia lineage using peripheral blood cell images. Comput. Methods Programs Biomed..

[35] Ma L., Cheng S., Shi Y. (2020). Enhancing learning efficiency of brainstorm optimization via orthogonal learning design. IEEE Trans. Syst. Man Cybern. Syst..

[36] Nguyen D., Long T., Jia X., Lu W., Gu X., Iqbal Z., Jiang S. (2019). A feasibility study for predicting optimal radiation therapy dose distributions of prostate cancer patients from patient anatomy using deep learning. Sci. Rep..

[37] Bertelli E., Mercatelli L., Marzi C., Pachetti E., Baccini M., Barucci A., Colantonio S., Gherardini L., Lattavo L., Pascali M.A. (2021). Machine and Deep Learning Prediction Of Prostate Cancer Aggressiveness Using Multiparametric MRI. Front. Oncol..

[38] Awotunde J.B., Chakraborty C., Adeniyi A.E. (2021). Intrusion detection in the industrial internet of things network based on deep learning model with rule-based feature selection. Wirel. Commun. Mob. Comput..

[39] Al-Antari M.A., Al-Masni M., Park S.-U., Park J., Metwally M.K., Kadah Y.M., Han S.-M., Kim T.-S. (2018). An automatic computer-aided diagnosis system for breast cancer in digital mammograms via deep belief network. J. Med. Biol. Eng..

[40] Ahmed L., Iqbal M.M., Aldabbas H., Khalid S., Saleem Y., Saeed S. (2020). Images data practices for semantic segmentation of breast cancer using deep neural network. J. Ambient. Intell. Humaniz. Comput..

[41] Mahmood T., Arsalan M., Owais M., Lee M.B., Park K.R. (2020). Artificial intelligence-based mitosis detection in breast cancer histopathology images using faster R-CNN and deep CNNs. J. Clin. Med..

[42] Awotunde J.B., Adeniyi A.E., Ajagbe S.A., Jimoh R.G., Bhoi A.K. (2022). Swarm Intelligence and Evolutionary Algorithms in Processing Healthcare Data. Connected e-Health.

[43] Awotunde J.B., Abiodun K.M., Adeniyi E.A., Folorunso S.O., Jimoh R.G. (2022). A deep learning-based intrusion detection technique for a secured IoMT system. Communications in Computer and Information Science.

[44] Ogundokun R.O., Awotunde J.B., Sadiku P., Adeniyi E.A., Abiodun M., Dauda O.I. (2021). An enhanced intrusion detection system using particle swarm optimization feature extraction technique. Procedia Comput. Sci..

[45] Casas S., Portalés C., Morillo P., Fernández M. (2018). A particle swarm approach for tuning washout algorithms in vehicle simulators. Appl. Soft Comput..

[46] Yang J., Zhang L., Chen C., Li Y., Li R., Wang G., Jiang S., Zeng Z. (2020). A hierarchical deep convolutional neural network and gated recurrent unit framework for structural damage detection. Inf. Sci..

[47] Fuchs A., Heider Y., Wang K., Sun W., Kaliske M. (2021). DNN2: A hyper-parameter reinforcement learning game for self-design of neural network-based elastoplastic constitutive descriptions. Comput. Struct..

[48] Medjahed S.A., Saadi T.A., Benyettou A., Ouali M. (2017). Kernel-based learning and feature selection analysis for cancer diagnosis. Appl. Soft Comput..

[49] Wang Y., Yang X.G., Lu Y. (2019). Informative gene selection for microarray classification via the adaptive elastic net with conditional mutual information. Appl. Math. Model..

[50] Alanni R., Hou J., Azzawi H., Xiang Y. (2019). A novel gene selection algorithm for cancer classification using microarray datasets. BMC Med. Genom..

[51] Salem H., Attiya G., El-Fishawy N. (2017). Classification of human cancer diseases by gene expression profiles. Appl. Soft Comput..

[52] Liu J., Wang X., Cheng Y., Zhang L. (2017). Tumor gene expression data classification via sample expansion-based deep learning. Oncotarget.

[53] Ram M., Najafi A., Shakeri M.T. (2017). Classification and biomarker genes selection for cancer gene expression data using random forest. Iran. J. Pathol..

[54] Ilievski I., Akhtar T., Feng J., Shoemaker C. (2017). Efficient hyperparameter optimization for deep learning algorithms using deterministic RBF surrogates. Proceedings of the AAAI Conference on Artificial Intelligence.

[55] Adeniyi E.A., Gbadamosi B., Awotunde J.B., Misra S., Sharma M.M., Oluranti J. (2021). Crude Oil Price Prediction Using Particle Swarm Optimization and Classification Algorithms. International Conference on Intelligent Systems Design and Applications.

[56] Eberhart R.C., Shi Y. (1998). Comparison between genetic algorithms and particle swarm optimization. International Conference on Evolutionary Programming.

[57] Gaing Z.L. (2003). Particle swarm optimization to solve the economic dispatch considering the generator constraints. IEEE Trans. Power Syst..

